# Magnetic Resonance-Guided Cancer Therapy Radiomics and Machine Learning Models for Response Prediction

**DOI:** 10.3390/tomography10090107

**Published:** 2024-09-02

**Authors:** Jesutofunmi Ayo Fajemisin, Glebys Gonzalez, Stephen A. Rosenberg, Ghanim Ullah, Gage Redler, Kujtim Latifi, Eduardo G. Moros, Issam El Naqa

**Affiliations:** 1 Department of Physics, University of South Florida, Tampa, FL 33620, USA; jesutofunmi.fajemisin@moffitt.org (J.A.F.); gullah@usf.edu (G.U.);; 2 Machine Learning Department, H. Lee Moffitt Cancer Center & Research Institute, Tampa, FL 33612, USA; glebys.gonzalez@moffitt.org; 3 Radiation Oncology Department, H. Lee Moffitt Cancer Center & Research Institute, Tampa, FL 33612, USA; gage.redler@moffitt.org (G.R.); kujtim.latifi@moffitt.org (K.L.)

**Keywords:** MRI, MRI-Linac, radiomics, clinical outcomes, machine learning

## Abstract

Magnetic resonance imaging (MRI) is known for its accurate soft tissue delineation of tumors and normal tissues. This development has significantly impacted the imaging and treatment of cancers. Radiomics is the process of extracting high-dimensional features from medical images. Several studies have shown that these extracted features may be used to build machine-learning models for the prediction of treatment outcomes of cancer patients. Various feature selection techniques and machine models interrogate the relevant radiomics features for predicting cancer treatment outcomes. This study aims to provide an overview of MRI radiomics features used in predicting clinical treatment outcomes with machine learning techniques. The review includes examples from different disease sites. It will also discuss the impact of magnetic field strength, sample size, and other characteristics on outcome prediction performance.

## 1. Introduction

Personalized cancer medicine is an emerging practice that focuses on using patient-specific physiological and molecular characteristics to assist decision-making concerning the prevention, diagnosis, and prognosis of cancer [1]. These patient-specific features can be acquired from medical images, which not only visualize tumor sites and organs at risk but also provide biological information and functional genomics [2]. This information promises improvement in early cancer detection and can help develop personalized therapies and prognostic classifications in the future.

There are different imaging modalities used for medical image acquisition: positron emission tomography (PET), magnetic resonance imaging (MRI), computed tomography (CT), ultrasound imaging, etc. These modalities have different modes of operation and applications in diagnosis and therapy. Magnetic resonance (MR) images are known for their soft tissue delineation between tumors and normal tissues and better contrast resolution when compared to other modalities [3]. In chemotherapy, MRI is a valuable tool for monitoring some breast cancer patient’s response to treatment by comparing the pre-and post-treatment images. It is also used in assessing residual tumors after mastectomy and guiding biopsies [4,5,6,7]. With the development of the combination of MRI and linear accelerators, MRI-Linacs, there has been an increased use of MRI in radiotherapy planning for accurate delineation of the gross tumor volume, GTV, and nearby critical organs at risk [8]. Furthermore, in MR-guided adaptive radiotherapy (MRgRT), MRI is used in real-time image guidance to monitor tumor motion and anatomical changes during treatment [9,10]. This enables the adaptation of treatment plans to the anatomy of the day, which in turn improves clinical outcomes [11]. The extensive biological information obtained during MRI acquisition leads to an excellent source of data/information for building computational models for response predictions [12].

Patient-specific image features can be extracted from MR images for potential cancer diagnosis, prognosis, and prediction of treatment outcomes through a method called radiomics [13]. Radiomics involves the extraction of quantitative image features that describe the heterogeneity and statistical distribution of a particular region of interest, providing information about its shape, size, phenotype, and texture [13,14]. These features may help differentiate this region from the rest of the image, have a potential relationship with cancer treatment outcomes, and present an opportunity to be used as a predictive biomarker for these outcomes [15].

This review’s first section focuses on the principles of radiomics and its workflow. The second section will focus on the overview of MRI radiomics, followed by a section on magnetic resonance-guided radiotherapy and the similarities and differences between higher and lower magnetic field strength MRI-Linacs. Lastly, the Discussion section explores the impact of magnetic fields, feature selection techniques, and other factors on outcome models for response prediction.

## 2. Radiomics Workflow

The difference in the physiology between tumor cells and surrounding healthy tissues is the basis for the need for medical imaging in cancer therapy. Radiomics involves the extraction of high-dimensional quantitative features, which may provide pathological information about the disease sites and potentially correlate with the clinical outcomes, aiding its application in diagnosis and prognosis [16].

**Figure 1:**
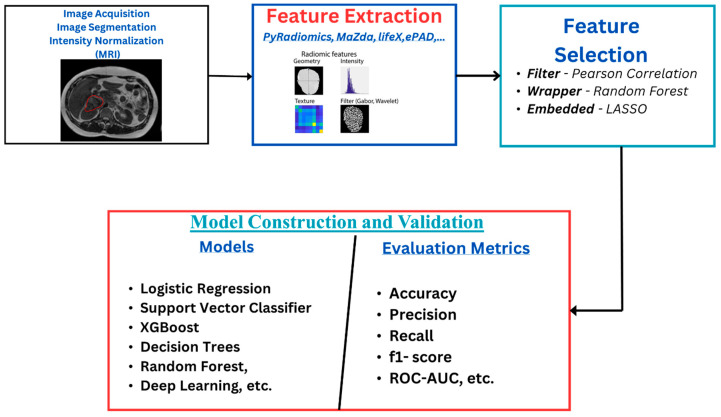
Radiomics overflow starts with image acquisition, which can be any imaging modality, such as MRI. This is followed by segmentation of the region of interest and the application of some preprocessing techniques like voxel resampling and intensity normalization. After this, features are extracted from the region of interest, which are used for statistical and machine learning analysis for clinical outcome model prediction.

The radiomics workflow (Figure 1) starts with image acquisition, which can be any of the imaging modalities—MRI, PET, CT, etc.—followed by the segmentation of the region of interest and the application of preprocessing techniques such as voxel resampling and intensity normalization. Next, features are extracted from the region of interest, which are used for statistical and machine learning analysis for clinical outcome model prediction [17,18].

Radiomics features reflect information about the tumor with respect to its size, shape, phenotype, texture, etc. [13,19]. Some features strongly correlate with the clinical outcomes, which aids in its application in diagnosis and prognosis. They are classified into first-order, second-order, and wavelet features. First-order features are statistical features that give information about the distribution of the voxel intensities without considering the spatial distribution. At the same time, second-order features analyze the spatial distribution of the image intensities and also the relationship between neighboring voxels. Features that are extracted when the images have undergone wavelet transformations are called wavelet features [19,20,21,22].

### 2.1. Feature Selection Techniques

The number of highly correlated features affects the quality of the radiomics model, which can lead to overfitting and poor generalization of the model. Improving the quality of the features involves distinguishing between the relevant features for a specific outcome prediction and the redundant/irrelevant ones. Care must be taken as some features have been identified as having intrinsic dependencies. In contrast, others may be affected by intensity discretization, spatial resolution, scanner variability, and even by the kernel used during image reconstruction [23,24,25]. This makes the feature selection stage of the radiomics workflow an essential process. Different techniques are reported in the literature, and authors have reported using one or a combination of various techniques to select the relevant features from these high-dimensional extracted features (see Supplementary Tables S1–S5). These techniques are categorized into three major groups—filter, wrapper, and embedded methods. In the filter method, features are selected based on a predefined threshold on the correlation coefficients or other statistical tests with respect to the target variable without considering any specific machine learning algorithm. In contrast, in the wrapper method, feature subsets are evaluated by training the machine learning algorithm on a different combination of features and selecting the subset that best optimizes the model’s performance, whereas, in the embedded method, features are selected during the machine learning model’s training. The model’s predictive performance and the relevance of selected features are optimized simultaneously [21].

### 2.2. Machine Learning Models for Response Prediction

After extraction and selection of relevant features, a machine learning (ML) model is used to classify patients into different groups according to the treatment outcome that is to be predicted. Here are some of the common ML models: logistic regression (LR), naïve Bayes (NB), decision trees (DT), random forest (RF), adaptive boosting (AdaBoost), extreme gradient boosting (XGBoost), and deep learning (DL).

## 3. MRI Radiomics Models for Response Prediction

### 3.1. Literature Review

**Figure 2:**
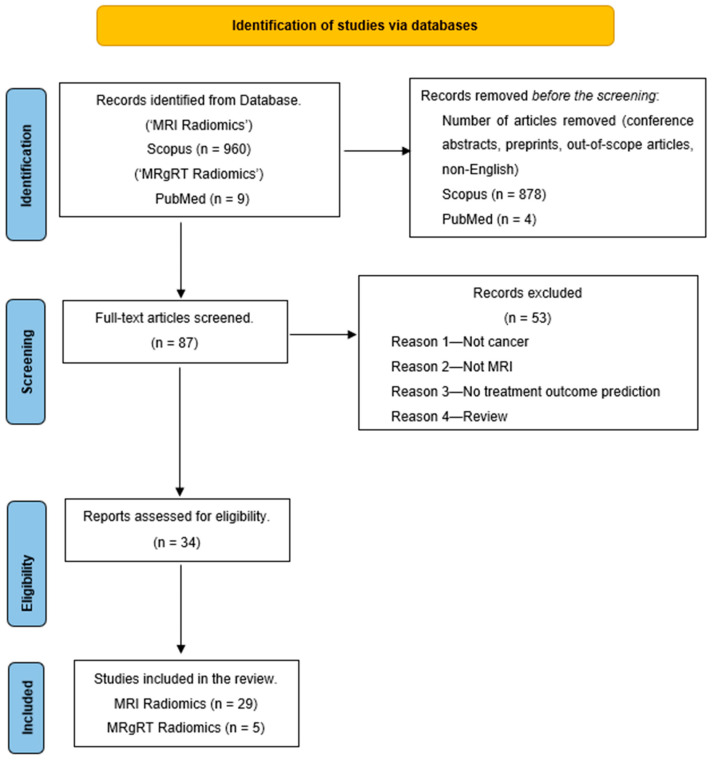
PRISMA outline of the review.

A literature search was conducted in December 2023 by searching for the keywords “MRI Radiomics AND Machine Learning” on the Scopus database. The search was limited to studies between 2012 and 2023 and only full-text articles in English. Also, an additional search for “Delta Radiomics MRgRT” on PubMed gave nine articles that fit this review’s purpose. The search result was narrowed to 82 articles on Scopus and five from PubMed. These 87 articles were further screened, and a few were excluded for various reasons, such as articles that were unrelated to treatment outcome prediction, not related to cancer, or were review articles. This reduced our articles to 34 for the literature review. Figure 2 shows the PRISMA diagram of the literature search workflow.

The goal of this review is to provide a lookup table that contains an overview of MRI (Supplementary Tables S1–S5) and MRgRT radiomics response prediction machine learning models—the image preprocessing techniques, feature selection techniques, machine learning algorithms, relevant features selected, and model evaluation results.

### 3.2. Overview of MRI Radiomics Models for Response Prediction

Patients’ responses to treatment can be determined by estimating different outcomes like progression, local/distant control, and overall survival. As mentioned earlier, radiomics features may describe the histology of tumor cells, their physiology, and microenvironment characteristics and are thought to be correlated to clinical outcomes as they relate to the tumor under observation (though they may also potentially impact distant metastases through a local process) [26]. Thus, radiomics may be leveraged to build prediction models for treatment response [27]. Radiomics models are computational models that use quantitative features from medical images to characterize and predict cancer treatment outcomes. After treatment, machine learning models have been used to predict the outcome/response to these treatments. Here are a few examples in the literature of how radiomics models have been used to predict treatment outcomes in various cancers such as glioblastoma, nasopharyngeal, cervical, hepatocellular carcinoma, and breast.

#### 3.2.1. Brain Cancer

Patel et al. [28] employed a machine-learning classification model for glioblastoma patients to predict true progression and pseudoprogression after chemotherapy. A total of 307 radiomics feature was extracted from contrast-enhanced T1-weighted images (CE-T1WI), T2-weighted images (T2WI), and Apparent Diffusion Coefficient (ADC) maps scanned on a 1.5 T MRI scanner. After feature selection, six feature models were investigated, consisting of different combinations of clinical, molecular, and radiomics features. The optimal model for the classification of the patients in the test set with an AUC of 0.80 (0.74–0.86) is composed of a clinical feature (Age), a molecular feature (MGMT methylation), and seven radiomics features (see Supplementary Table S1).

Ammari et al. [29] developed a machine learning-based radiomics MRI model to predict overall survival (OS) and progression-free survival (PFS) in glioblastoma patients treated with bevacizumab. A cohort of 194 patients was divided into training and testing sets for survival regression, 9-month survival, 12-month survival, 15-month survival, 6-month progression, and 12-month progression models. Seven classification models were trained—random forest, gradient boosting, AdaBoost, logistic regression (LR), k-nearest neighbor (KNN), naïve Bayes (NB), and support vector machine (SVM). For 9-month OS, LR has an AUC of 0.78; for 12-month OS, SVM has an AUC of 0.85; for 15-month OS, RF has an AUC of 0.76; and PFS of 6 months has an AUC of 0.71 on the test set (see Supplementary Table S1).

#### 3.2.2. Nasopharyngeal Carcinoma

Du et al. [30] presented a two-center study to predict the 3-year disease progression of non-metastatic nasopharyngeal carcinoma after intensity-modulated radiation therapy. There were 277 patients from two institutions with CE-T1WI and T2WI with a 3.0 T MRI scanner. Five hundred and twenty-five features were extracted per patient. Pearson correlation coefficient, intraclass coefficient, and hierarchical clustering were used to select four radiomic features in combination with clinical features like the T stage and overall stage. The support vector machine has an AUC of 0.80 on the test set (see Supplementary Table S2).

#### 3.2.3. Liver Cancer

Chen et al. [31] predicted the response to transarterial chemoembolization in hepatocellular carcinoma patients. One hundred and forty-four patients were randomly assigned to training and test sets. Four hundred and forty features were extracted from axial T2-weighted and mDIXON-T1WI images from a 1.5 T or 3.0 T scanner. Minimum redundancy maximum relevance selection was used for dimensionality reduction in KNN and SVM, while LASSO and deep neural networks do not require feature selection. The deep neural network outperformed other models in the test set, and the clinical model and DNN model achieved an AUC of 0.831 on the test set and an AUC of 0.735 on external validation (see Supplementary Table S3).

#### 3.2.4. Breast Cancer

Chen et al. [32] built a machine learning-based radiomics nomogram to predict neoadjuvant chemotherapy efficacy in breast cancer patients. Maximum relevance minimum redundancy and LASSO selection were used to reduce features from 256 to 6 optimal features. The radiomics signature gave an AUC of 0.834 on the test set (see Supplementary Table S4).

#### 3.2.5. Other Cancer Sites

In cervical cancer, Jajodia et al. [33] used a cohort of 52 patients with various FIGO stages are used in this study. Eight hundred and fifty-one features were extracted from DWI, ADC maps, and T2WI images from a 3 T scanner. The Pearson correlation coefficient feature selection technique was used. Multiple models were used to predict recurrence distant metastasis, lymph node metastasis, and Figo stage (see Supplementary Table S5).

In endometrial cancer, Lin et al. [34] developed a fusion model based on the clinicopathological factors and MRI radiomics features to predict recurrence risk in patients with endometrial cancer. One thousand seven hundred and two radiomics were extracted from a 1.5 T or 3.0 T scanner. A total of 337 patients from a center (235 training, 102 validation) were used for the internal training of the model. In comparison, a combination of 84 patients from three other institutes was used for external validation. One thousand and seventy-two features were extracted from T2WI and CE-T1WI images. One-way ANOVA and LASSO were used for feature selection, and the XGBoost classifier was used for the classification model. The best model was the fusion model based on the intertumoral area, which had the optimal performance in predicting recurrence risk (see Supplementary Table S5).

## 4. Magnetic Resonance for Image-Guided Radiotherapy

Image guidance during radiotherapy has helped improve the appropriate delivery of radiotherapy to tumor cells. The visualization of the GTV and, in some cases, accounting for its motion helps control dose delivery to healthy tissues [11]. There has been a growth in the use of MR-guided Radiotherapy (MRgRT), in which linear accelerator systems are combined with MRI scans. With this technology, an MRI may be obtained each day for radiotherapy delivery, and it has been reported to provide superior visibility of the organs (particularly in gastrointestinal traction) and allow for the opportunity to decrease margins and adaptive therapy [35]. This has helped the implementation of modifying on-table RT treatment plans to consider daily anatomic changes while administering ablative doses to the target and managing respiratory movements with cine images [36].

Rudra et al. [37] reported a retrospective observational study on adaptive MRgRT across five institutions for treating patients with inoperable pancreatic cancer with a ViewRay 0.35 T MRI-Linac. They reported an improved overall survival for patients treated with dose-escalated MRgRT. Parikh et al. [38] conducted a phase 2 evaluation study on the safety of 5-fraction Stereotactic MR-guided on-table Adaptive Radiation Therapy (SMART) for locally advanced and borderline resectable pancreatic cancer. Among 136 patients treated, no acute grade ≥ 3 gastrointestinal (GI) toxicity was definitively attributed to SMART, meeting the primary endpoint. However, 8.8% experienced potential SMART-related acute grade ≥ 3 GI toxicity, including two postoperative deaths. The one-year overall survival rate was 65.0%. Therefore, for patients with localized tumors in the stomach region who are not surgical candidates, stereotactic MRgRT may be a feasible and safe non-invasive treatment option that results in minimal impact on the sensitive organs [35,39].

Two major MRI-Linacs are commercially available for treatment in the United States —high-field (1.5 Tesla) MRI-Linac [40] and low-field (0.35 T) [41]. Engineering solutions have been implemented to accommodate an MRI in a linac environment and vice versa. Liney et al. [36] reviewed the challenges encountered in developing this type of technology. Table 1 shows the differences and similarities between the two designs with respect to the measures taken to accommodate an MRI scanner in a linac environment and a linac in an MRI environment [8,36,40,41,42,43,44,45].

### Delta Radiomics and MRgRT Radiomics Models for Response Predictions

The study of the effects of radiomics feature variations at different acquisition times in the patients’ treatment workflow, either prior, during, or after treatment, is called Delta Radiomics. These variations can be calculated as the difference between features from images before and after treatment or the ratio of features from a particular fraction to the first or simulation fraction.

In chemotherapy, delta radiomics features can be defined as the difference between the features extracted from the pre-treatment and post-treatment images [46]. Chang et al. [47] and Peng et al. [48] defined their delta features as the ratio of post-treatment to pre-treatment features. These features were reported to improve the predicting power of the model. In the case of Peng et al., the combination of the delta features and pre-treatment features improved the AUC of the KNN model to 0.90 (95% CI: 0.848–0.956).

Tomaszewski et al. [49] presented a delta radiomics analysis in MRgRT to predict progression-free survival in pancreatic adenocarcinoma by using the ratio of the last fraction, F5, to that of the first fraction, F1. It was reported that there is a significant association between histogram skewness change during treatment and progression-free survival. Cusumano et al. [50] also reported that a delta radiomics analysis that changes in cluster shade at a biologically equivalent dose (BED) of 40 Gy can predict one-year local control for patients with locally advanced pancreatic cancer treated with magnetic resonance-guided radiotherapy. Boldrini et al. [51] reported two significant (*p* = 0.001) delta radiomics features as predictors of clinical complete response (CR) after neoadjuvant radio-chemotherapy in locally advanced rectal cancer patients. The delta features are the variations in the smallest axis length, ΔL_least,_ and grayscale nonuniformity, Δglnu, at BED = 26.8 Gy to the value calculated on the simulation MR. On external validation, Cusumano et al. [52] reported that ΔL_least_ accurately identified patients with cCR and pCR 35 and 33 patients, respectively. Table 2 shows an overview of the MRgRT delta radiomics literature review.

## 5. Quality of Radiomics Model

When developing radiomics models, the initial selection of the most relevant features is crucial to producing a model that will correctly generalize unseen data. Radiomics inherently presents a challenge for feature selection since many features are correlated. Additionally, the initial quality of the image is affected by the MRI acquisition parameters.

Feature robustness is defined by its repeatability under similar imaging conditions and its reproducibility under diverse imaging conditions. For CT radiomics, our group, Shafiq-ul-Hassan et al. [24] reported the variation in CT phantom radiomic features on voxel size and number of gray levels. The study examined the impact of slice thickness, pixel spacing, and gray-level discretization on radiomics features extracted from CT phantom images. It focuses on the reproducibility of these features across different scanners and varying acquisition and reconstruction parameters. A texture phantom with 10 different cartridges was scanned on eight CT scanners, and 213 radiomics features were extracted. The study included voxel-size resampling of image sets and feature extraction from both original and resampled datasets. The results showed that 150 of the 213 features were reproducible across voxel sizes, 42 improved significantly after resampling, and 21 had large variations before and after resampling. Ten features significantly improved after modifying definitions to remove voxel-size dependency, with interscanner variability nearly vanishing for eight of these features. Redefining texture features to include gray levels significantly reduced dependency. The study concludes that voxel-size resampling is effective for achieving more reproducible CT features across datasets with variable voxel sizes.

Another study by Shafiq-ul-Hassan et al. [23] investigated the impact of pitch, dose, and reconstruction kernel on CT radiomic features, finding that most texture features were dose-independent but strongly kernel-dependent. The ACR phantom for 3D noise power spectrum (NPS) measurements and applied NPS peak frequency and ROI maximum intensity were used as correction factors to reduce variability. They reported that these corrections significantly improved the robustness of 19 features by 30% to 78%, demonstrating that NPS peak frequency and ROI maximum intensity effectively mitigate the variability in CT texture feature values due to reconstruction kernels.

Few studies have been reported on the robustness of MRI radiomic features. The section below discusses the effect of magnetic fields on radiomic features.

### Effect of Magnetic Field on Radiomics Features

The quality of a radiomics model is dependent on the reproducibility of the relevant features as biomarkers for a disease site or clinical outcome [54]. Unfortunately, these features are sensitive to image quality, which is dependent on MRI acquisition parameters like magnetic field strength, image acquisition parameters, sequences, pixel size, and signal-to-noise ratio [55,56]. At higher magnetic fields, image quality increases, leading to high SNR, thus increasing spatial and temporal resolution. The cons remain that achieving static uniform magnetic fields becomes difficult, and inhomogeneities in the field introduce artifacts in the images, which are more prominent in higher fields than in lower fields [57,58,59,60].

Our group, Ericsson-Szecsenyi et al. [25] studied the variability and identified highly repeatable and reproducible radiomics features from images acquired with a 0.35 T MRI-Linac scanner. We analyzed eleven scans of each of the Magphan^®^ RT and ViewRay Daily QA phantoms and 50 images from ten anonymized SBRT pancreatic cancer patients, using a TRUFI pulse sequence with specific voxel resolutions. They extracted 1087 shape-based, first-, second-, and higher-order features, followed by a robustness analysis using the coefficient of variation (CoV < 5%). The study identified 130 robust features across the datasets, though none from the GLSZM and NGTDM second-order sub-groups. Several of these robust features were consistent with findings from other stability assessments and predictive performance in the literature. We concluded that the 0.35 T scanner is stable for longitudinal radiomics phantom studies and that phantom measurements can effectively identify robust radiomics features. We emphasized the need for further stability assessment research.

Ammari et al. [61] reported the influence of magnetic field strength on the texture features in neuroradiology clinical practice. They evaluated the impact of the field (1.5 T, 3.0 T) on radiomics features from the same manufacturer. Statistical differences between 1.5 and 3.0 T features were determined by Student’s *t*-test using paired data. Thirty-eight features were extracted in the following categories: intensity histogram and texture features. Most texture feature values were significantly different on homogenous phantoms, e.g., the entropy mean value differed by a factor of 4. In heterogeneous phantoms, histogram features like kurtosis, entropy, and energy and matrix features like Low Zone High Gray Energy showed no significant difference. On healthy volunteers, 15 out of 38 features showed significant differences.

Also, Cusumano et al. [62] reported that selecting appropriate image features can help overcome the effect of the variability of the magnetic field in the radiomics model. This study used two datasets from two machines of different magnetic field strengths (1.5 T vs. 3.0 T). Three of the 486 extracted features were selected for building a multivariate logistic regression model to predict pathological complete response in locally advanced rectal cancer. This model was trained on the whole dataset and tested on the individual data, and the AUC of the model on the combined data was 0.72, while the values of 0.70 and 0.83 on the 1.5 T and 3.0 T, respectively. The higher AUC of the 3.0 T model results from high SNR in the images and higher spatial resolution.

The variation in radiomics features with respect to changes in the magnetic field strength of the scanner should be considered for future analysis. For cases with datasets from multiple scanners with different magnetic field strengths, caution must be taken to ensure that the selected features for model building are independent of the field strength.

## 6. Discussion

MRI is commonly used to image diverse regions of the body. Its precise soft-tissue contrast offers a database for the use of radiomics models to predict treatment outcomes for these sites of the body. Machine learning-based radiomics models learn the underlying relationships between the extracted features and the treatment outcomes. This helps to improve personalized outcome prediction based on the patient-specific features extracted from patients’ MRIs. The radiomics model aims to provide decision support for personalized treatment.

This review has presented examples of models from different disease sites with varying clinical outcomes. These models are built using different radiomic features and in combination with clinicopathological features. The quality of these models is affected by factors like sample size, model-building algorithm, and level of correlation within selected features. In contrast, the quality of the selected features is affected by the feature selection techniques and the image quality with respect to the MRI scanner acquisition parameters like magnetic field and sequences.

***Effect of Training Sample Size:*** This significantly impacts how well radiomics models perform. Models trained on a larger dataset are more stable and reliable. When applied to an unseen or external dataset, variations and outliers in the training data can affect a small-sample-size model’s performance. The likelihood of overfitting and poor model generalization is increased. From the literature in the Supplementary Tables, the sample sizes in Shahveranova et al. [63] and Cepeda et al. [64] are 42 and 45, respectively. Very few studies in this review have sample sizes above 200. Overcoming the effect of small sample sizes can be achieved through multicenter datasets, as reported by Du et al. and others [30,34,64,65,66]. Also, using a publicly available dataset is another approach reported by Ammari et al. [29] and Suter et al. [66] They used a public dataset called the BraTS challenge data for training and validation. After training with institutional data, Suter et al. reported a poor validation of the model on the public data with an AUC of 0.56. Ammari et al. reported a better performance when the model was trained on the BraTS dataset and validated the model with the institutional data. Using multi-center datasets is highly recommended to improve the robustness of the model.

***Effect of ML algorithms:*** The accuracy, interpretability, generalization, and computational efficiency of the radiomics model are all influenced by the machine learning model selection. Different models excel in different scenarios depending on the nature of the data, correlations between selected features, level of interpretability, and proposed clinical application. It is widely acknowledged that several machine learning models’ level of performance should be compared, and the model that makes the best predictions and/or the most interpretable model is recommended. Almost all the studies in this review compared results from multiple ML models, and the model with the best performance was selected. We recommend that the explainability of the models should be explored for their easy clinical implementation in the future. The most commonly used ML models are logistic regression (LR), support vector machine (SVM), k-nearest neighbor (KNN), random forest (RF), extreme gradient boosting, and deep learning models.

***Effect of Magnetic Fields:*** Higher magnetic field strengths may enhance image quality, but they may additionally introduce artifacts in the images. Improved image quality implies a high signal-to-noise ratio and high tissue contrast, which affect the values of texture- and intensity-based features. Unfortunately, the reliability of these features is affected by the prominent artifacts that are associated with images from higher fields. The variability in the magnetic field strength across different MRI scanners or imaging protocols can lead to inconsistencies in radiomics features extracted from different datasets, affecting their reproducibility and generalizability. As explained earlier in the research conducted by Ammari et al. and Cusumano et al., some texture features are magnetic field strength-dependent, which could affect the generalizability of such a radiomics model, except if it comprises robust features that are independent of the magnetic field of the scanner [61,62]. This would promote multi-institutional radiomics projects.

***Effect of Feature Selection Techniques:*** Removing irrelevant or redundant features can improve the performance of radiomics models. Feature selection helps to mitigate overfitting by selecting a subset of features that capture the underlying patterns in the data while reducing the risk of fitting highly correlated features into the model. Reducing the dimensionality of the feature space is much needed when the sample size (N) is far less than the number of features (k) to identify features that better represent the relationship between the features and the clinical outcome to build models that are more robust and generalizable across different datasets and image acquisition parameters.

## 7. Conclusions

The application of radiomics offers a powerful approach for extracting quantitative information from MRI images, which can provide insight into tumor biology, tumor response to treatment, and patient outcomes, ultimately contributing to improved diagnosis and prognosis in oncology. The quality of the model can be improved by selecting features that are more robust and reproducible across different datasets and image acquisition parameters. The future direction of multi-institutional MRI radiomics research is to study the effect of magnetic field strengths on the quality of radiomic features extracted from cancer sites. This will help to establish conditions of robustness and harmonization of patient-specific features across multi-institutional MRI acquisition parameters.

## Figures and Tables

**Table 1:** The differences and similarities between the two designs with respect to the measures taken to accommodate an MRI scanner in a linac environment and vice versa. This difference is deducted from the ICRU REPORT 97 [45].

	High-Field Design	Low-Field Design
**Radiation source**	7 MV Flattening Filter Free.	6 MV Flattening Filter Free
**Magnetic field strength**	1.5 T	0.35 T
**Magnet orientation**	Closed superconducting. The radiation beam is perpendicular to the magnetic field, Bo.	Split superconducting. The radiation beam is perpendicular to the magnetic field, Bo.
**Linac in the MRI Environment**		
**RF power source (Magnetron) in B-field**	Magnetron rotates with the linac and is positioned to sit in the low-magnetic-field region.	
**Waveguide design**	Short waveguide design with no bending magnet.	
**The angle of radiation delivery without significant beam perturbation**	Accelerate through cryostat. The exclusion zone depends on the target location to guarantee that no portion of the beam penetrates via the cryostat pipe.	There is no full gantry motion. It cannot rotate between 30° and 33°.
**Motors—collimator, MLC, gantry, Couch in B-field**	The superconducting coil’s arrangement is adjusted to create a low-intensity toroidal magnetic field, ensuring the optimal positioning of the most sensitive linac component.	The linac-sensitive components are isolated on a gantry ring and housed within shielded cylindrical baskets.
**MRI Scanner in the Linac Environment**		
**Effect of RF power source and motors on image noise**	Use of a Faraday cage to separate the electrically noisy components from the MRI environment.	A radiofrequency cage around the linac and MRI components individually.
**Effect of gantry rotation, moving jaws, and MLC on Bo homogeneity**	Passive shimming. Active shimming.	Gantry angle-specific active shimming.

**Table 2:** MRgRT delta radiomics response prediction model literature review.

First Author	Cancer Site	No of Centers	Sample Size	Treatment Modality	Outcomes	MRI-Linac (Magnetic Field)	Radiomics/Delta Features Extracted	Features Used in Modeling	Prediction Model Assessment	Model Evaluation Results
Boldrini et al., 2021 [51,52]	Rectal Cancer	3	59 Training = 16 Testing = 43	Neoadjuvant radiochemotherapy	Clinical complete response, nCR Partial response, pCR	0.35 T MRI-Linac TRUFI sequence	318 features Delta features = ratio of features at BED = 26.8 Gy to the simulation fraction.	ΔGray level non-uniformity, Δglnu ΔLeast axis length, ΔL_least_	ROC curve analysis Youden Index	Training Data ΔL_Least_ AUC = 0.82 for cCR and 0.93 for pCR Δglnu AUC = 0.72 for cCR and 0.54 for pCR External Validation ΔL_Least_ = 0.81 for cCR and 0.71 for pCR Δglnu = 0.63 for cCR and 0.40 for pCR
Cusumano et al., 2021 [50]	Pancreatic Cancer	2	35	MRgRT	One-year local control	0.35 T MRI-Linac TRUFI Sequence	644 features	**Most significant feature** GLCM variation of cluster shade (*p*-value = 0.005)	ROC curve analysis	Cross-validation AUC = 0.79 (95% CI = 0.62–0.97)
Tomaszewski et al., 2021 [49]	Pancreatic Cancer	1	26	MRgRT	PFS	0.35 T MRI-Linac TRUFI Sequence	73 features Delta features = F5/F1	Histogram Skewness (Hazard Ratio 2.75 (1.36–5.56) *p* = 0.038		
Wu et al., 2023 [53]	Rectal Cancer	1	28	MRgRT	Pathological Complete Response, pCR Clinical Complete Response, cCR	1.5 T MRI-Linac	2324 features Delta features ΔF_i_ = F_i_/F_1_ F_i_ = features from MRI of ith fraction	**Clinical:** N-stage **Radiomics:** F1_GLZM Zone Entropy **Delta Radiomics:** ΔF2_GLSZM_Gray-level_variance, ΔF2_GLSZM_High_gray_level_zone_emphasis, ΔF2_GLSZM_Small_area_high_gray_level_emphasis, ΔF2_First_order_Range, ΔF2_GLSZM_gray_level_nonuniformity.	Rad Score LASSO Regression	These features significantly discriminate between pCR and non-pCR patients (*p* < 0.05)

Abbreviations: AUC = area under the curve; GLCM = gray-level cooccurrence matrix; GLZM = gray-level zone matrix; GLSZM = gray level size zone matrix; LASSO = least shrinkage selection operator; MRgRT = magnetic resonance-guided radiotherapy.

## Data Availability

No new data were created or analyzed in this study. Data sharing is not applicable to this article.

## Supplementary Materials

The following supporting information can be downloaded at: https://www.mdpi.com/article/10.3390/tomography10090107/s1, Table S1: Brain Cancer [28,29,47,64,65,66,67,68,69,70,71,72,73]; Table S2: Nasopharyngeal carcinoma [30,74,75]; Table S3: Breast Cancer [31,63,76,77]; Table S4: Breast Cancer [78]; Table S5: Other Cancer sites [33,34,79,80,81,82,83,84].

## Abbreviations

AUC	Area Under receiver operating Curve
ADC	Apparent Diffusion Coefficient
iAUC	incremental Area Under the Curve
CE-T1WI	Contrast Enhanced T1-weighted image
DCE	Dynamic Contrast Enhanced
DWI	Diffusion Weighted Image
DT	Decision Trees
GLCM	Gray Level Co-occurrence Matrix
GLDM	Gray Level Difference Matrix
GLSZM	Gray Level Zone Matrix
GLSZM	Gray Level Size Zone Matrix
GLZLM	Gray Level Zone Length Matrix
GLRLM	Gray Level Run Length Matrix
HR	Hazard Ratio
NTZ	Nitazoxanide
ICC	Intraclass Correlation Coefficient
IMRT	Intensity Modulated Radiation Therapy
KNN	K Nearest Neighbor
LASSO	Least Absolute Shrinkage and Selection Operator
LC	Local Control
LF	Local Failure
LR	Logistic Regression
MRI	Magnetic Resonance Imaging
mRMR	maximum Relevance Minimum Redundancy
MI	Mutual Information
NB	Naïve Bayes
NGTDM	Neighborhood Gray Tone Difference Matrix
OS	Overall Survival
PCC	Pearson Correlation Coefficient
PFS	Progression-Free Survival
RF	Random Forest
ROC	Receivers Operating Curve
SVM	Support Vector Machine
T1WI	T1-Weighted Image
T2WI	T2-Weighted Image
wavelet-H	High pass filter
wavelet-L	Low pass filter
