# Supplementary material for: Magnetic Resonance-Guided Cancer Therapy Radiomics and Machine Learning Models for Response Prediction
Source: Tomography. 2024 Sep 2;10(9):1439–54. doi: 10.3390/tomography10090107 (PMC11435563; doi:10.3390/tomography10090107)
Supplement: Supplementary file 1 [file tomography-10-00107-s001.zip › tomography-3173119-supplementary.pdf]

Supplementary Material - Literature Review of MRI Radiomics across different disease sites with varying outcomes.

Table S1: Brain Cancer

First Author	Cancer site	No of centers	Sample size	Treatment modality	Outcomes	MRI (Magnetic Field)	Image Preprocessing	Radiomics features extractor software; no of features	Feature selection method	Features used	Predictive Model	Model Evaluation results
Patel et.al 2021 [28]	Glioblastoma	1	76	Chemoradiotherapy	Early true progression, pseudoprogression	1.5T MRI<br><br>CE-T1WI, T2WI, DWI	Image Re-sampling, Intensity Normalization	Pyradiomics<br><br>307 features	PCC, Recursive feature elimination with a random forest classifier	**Clinical** – Age<br>**Molecular** – MGMT Methylation<br>**Radiomics** – CE-T1WI Sphericity, ADC_first_order_Kurtosis, ADC_GLCM_Correlation, ADC_NGTDM_Contrast, T2WI_GLDM Dependence Entropy	Naive Bayes Classifier	Overall AUC across fivefold validation = 0.80 (95% CI: 0.74-0.86)
Ammari et al. 2021 [29]	Glioblastoma	1	194	Chemotherapy - Bevacizumab	OS<br>PFS<br><br>**Three classes**<br>9 months<br>12 months<br>15 months	Two MRI scanners<br><br>1.5T & 3.0T<br><br>Pre-contrast& post-contrast T1WI<br>T2WI<br>FLAIR<br>DWI	Z-score normalization<br><br>spatial resampling (voxel size 1x1x1 mm^3)<br><br>Fixed Bin size of 37	Olea Sphere<br><br>109 features	C-index Covariance	**Radiomics**<br>FLAIR_Original first-order_10^{th} percentile, Gadolium_original_shape_sphericity, FLAIR_Area_to_volume_ratio, FLAIR_NGTDM texture, FLAIR_NGTDM_strength<br><br>**Clinical**<br>Deficit, Symptoms, Delay_R	Random Forest<br>Gradient Boosting<br>Adaboost, Logistic Regression<br>K-neighbors<br>Naïve Bayes<br>SVM	**AUC score on the test**<br><br>**OS**<br>**9 months**<br>Logistic regression<br>AUC = 0.78<br><br>**12 months**<br>SVM<br>AUC = 0.85<br><br>**15 months**<br>RFT<br>AUC = 0.76<br><br>**PFS**<br>Regression model<br><br>**6 months** AUC = 0.56<br>**9 months** AUC = 0.69
Ammari et al. 2021 [67]	Glioblastoma	2<br><br>Public data (2019 BraTS Challenge) and	BraTS challenge – 210<br><br>Validation – 116	OS<br><br>**BraTS**<br>9 months<br>12 months<br>15 months<br><br>**Private data**	Chemotherapy, chemo-radiotherapy	Two MRI Scanner<br><br>1.5 T<br>3.0T<br><br>Pre-contrast 3DT1WI, post-contrast	Pixel-wise normalization Co-registration (BraTS : FLAIR + CE-T1WI +mask; Val : CE-	Pyradiomics<br><br>448 features	Concordance Index	**Submask NCT/NET/ED -** Gadolinium _Sphericity, Flair_Minimum, Flair_strength, Gadolinium_strength, Flair_contrast, Flair_coarseness<br><br>**Submask ED-** Gadolinium_Sphericity,Flair_major axis length	KNN, random forest, logistic regression, gradient boosting, AdaBoost, Naïve Bayes, SVM	AUC on Test<br><br>**9 months**<br>RFT AUC = 0.85<br><br>**12 months**<br>RFT<br>AUC = 0.74

		institutional data		Median OS of 22 months		3DT1WI, T2-FLAIR	T1WI + FLAIR)<br>Skull-scrapping			**Clinical -** Age		**15 months**<br>Logistic Regression<br>AUC = 0.58<br><br>**22 months Val**<br><br>Logistic regression<br>AUC = 0.75
Chang et al. 2019 [47]	Malignant gliomas	1	12	Overall Survival	Concurrent stereotactic radiosurgery and Bevacizumab	1.5 T MRI<br><br>Pre-treatment T1WI, one-week post1 -treat, two-month post-treat T1W1, T2-FLAIR	Post 1 and post 2 were registered on the pre-treatment images.<br><br>FBN 64 bins	In-house MATLAB 2017a code<br><br>61 features<br><br>F_{pre}, F_{post1}, F_{post2}<br><br>Delta features<br><br>$\Delta F1 = (F_{post1} - F_{pre})/F_{pre}$<br><br>$\Delta F2 = (F_{post2} - F_{pre})/F_{pre}$	Univariate Cox regression<br><br>Out-of-bag permutation random forest<br><br>Three-layer back propagation neural network.<br><br>L1-regularized logistic regression.	**RF + KSVM Model**<br>T1_GLCM_Correlation, T1_GLSZM_Variation_of_Intensity, T1_ GLSZM_SmallSizeHigh_LevelEmphasis, T1_GLSZM_HighGrayLevelEmphasis T1_GLSZM_LowGrayLevelSizeEmphasis, T1_GLCM_ClusterShade, T1_GLRLM_GrayLevelNonUniformity, T1_GLRLM_RunLengthNonUniformity, T1_GLSZM_SizeZoneNonUniformity, T1_GLSZM_LargeSizeLowGrayLevelEmphasis<br><br>**RF +NN Model**<br>T1_GLCM_Correlation, T1_GLSZM_Variation_of_intensity, T1_GLSZM_SmallSizeHighGrayLevelEmphasis, T1_GLRLM_GrayLevelNonIniformity, T1_GLSZM_LargeSizeLowGrayLevelEmphasis	RF<br><br>L1-regularized Logistic regression<br><br>L2-regularized Logistic regression<br><br>Linear SVM<br><br>Kernel SVM<br><br>Three-layer back propagation NN.<br><br>Naïve Bayes	Delta features provide better treatment assessment than single time-point features.
Jaberipour et al. 2021 [68]	Brain metastasis	1	120	Hypo-fractionated SRT	Local control/ local failure	1.5 T MRI<br><br>CE-T1WI<br>T2-FLAIR	Resamples 0.5 x 0.5 x 0.5 mm^{3}.<br><br>Skull scrapping	Pyradiomics<br><br>800 features	PCC, mRMR, Sequential forward feature selection	**Radiomics**<br>Elongation Tumor<br>T1- GLDM GLN Edema<br>T2 GLDM- HGLE- Tumor<br>T1- GLSZM LAHGLE- Edema<br><br>**Clinical**<br>Previous WBRT, Targeted Systemic Treatment TD, Histology	KNN	**Radiomic Model**<br>**AUC** = 0.87<br>**Clinical + Radiomics Model**<br>**AUC** = 0.65
Du et al. 2023 [65]	Brain metastasis	2	N1 = 337<br>N2= 247<br>N3= 60<br>N4 =30	Stereotactic radiosurgery	Local failure	1.5T MRI	Resampling registration Normalization	Pyradiomics<br>2153 features	Correlation coefficient, LASSO, mRMR	**Clinical**<br>Age<br>Karnofsky Performance Status (KPS)	Gaussian Naïve Bates, KNN, RF, adaptive Boosting, SVN with linear	SVM<br>Clinical-radiomics<br>AUC = 0.93

						Axial CE<br>T1WI, T2WI,<br>DWI				Graded Prognostic Assessment (GPA) scores.<br>Number of metastases<br><br>**Radiomics – 223 features**	kernel Multilayer perceptron	Radiomics AUC = 0.90
Karami et al. 2019 [69]	Brain Metastasis	1	100	SRT	Local Control<br><br>Local Failure	1.5 T MRI<br><br>GCE T1WI, T2WI	Linear Interpolation resampling 0.5 X 0.5x 0.5 mm^3	MATLAB<br><br>3072 features	PCC<br><br>Mann-Whitney U test (p-value)<br><br>Forward feature selection AUC.632+	**Overall LC/LF**<br>Lesion-Margin_Wavelet_ILHL_GLCM_MaximumProbability_T2<br>Edema_Wavelet_IHHL_Histogram_Maximum_T2<br>Tumor_Histogram_Minimum_T2<br>Tumor-Margin _Wavelet_IHLH_Histogram_Minimum_T2<br>Lesion-Margin _Wavelet_IHLL_Histogram_Range_T2<br><br>**6-Month LC/LF**<br>Tumor-Margin _Wavelet_ILHL_GLCM_MaximumProbability_T2<br>Tumor-Margin _Wavelet_IHLH_Histogram_Minimum_T1<br>Edema_LBP_Median_T1<br>Tumour_LBP_Median_T1<br><br>**12-Month LC/LF**<br>Lesion-Margin_Wavelet_IHLL_GLCM_MaximumProbability_T2<br>Edema_Wavelet_IHLH_Histogram_Maximum_T2<br>Edema_Wavelet_IHLH_Histogram_MeanAbsoluteDeviation_T2<br>Tumor-Margin _Geometric_Convexity_T1	SVM	***AUC*.632+ (Optimal qMRI Biomarker)**<br><br>Overall, LC/LF AUC = 0.79<br><br>6-Month LC/LF AUC = 0.80<br><br>12-Month LC/LF AUC = 0.81
Suter et al. 2020 [66]	Glioblastoma	2<br><br>Train set – Private Test set - BraTS	N1 = 63<br><br>N2 = 76	Temozolomide-based chemoradiation	Overall survival	MRI	Resampling to 1mm isovoxels<br><br>Skull scrapping<br><br>Registration to CE-T1WI	Pyradiomics<br><br>265,604 features	ReliefF<br><br>Fisher Score<br><br>Gini index<br><br>Chi-Square score<br>joint mutual information	564 features<br><br>558-deep GLSZM features<br>Four shape features	KNN, SVC with linear and radial basis function RBF kernels, Gaussian processes, decision trees, random forest, multilayer perceptron, AdaBoost, naïve	AUC drop of 0.56 on the BraTS dataset

							Bias field correction		Conditional Infomax feature extraction.<br><br>double input symmetric relevance<br><br>mutual information maximization conditional mutual information<br><br>Interaction capping<br><br>t-test score<br><br>mRMR<br><br>mutual information feature selection		Bayes, quadratic discriminant analysis QDA, XGBoost, Logistic Regression	
Cepeda et al. 2023 [64]	Glioblastomas	5	55<br>Training = 40<br>Testing =15	Adjuvant treatment with temozolomide and radiotherapy after surgery	Local Recurrence regions	1.5 T<br>3 T<br>3 T<br>1.5 T<br>3 T<br>MRIs<br><br>T1WI, T2WI, FLAIR, T1WI contrast enhance.<br>ADC maps	z-score normalization resampling $1x1x1mm^3$	Pyradiomics<br><br>4730 features		19 first-order statistical features<br>75 texture features	RF, XGBoost, CatBoost, LightGBM	CatBoost region-based evaluation<br><br>AUC 0.81±0.09
Sun et al. 2021 [70]	Glioblastoma	1	77	Gross total or subtotal resection CCRT with TMZ, 6 cycles adjuvant TMZ after surgery	True Progression<br><br>Pseudo progression	MRI 3T<br><br>axial<br>T1W1 and T2WI,<br>FLAIR<br>CE-T1WI	Normalization	Analysis Kinetics<br><br>9675 features	PCC<br><br>RF feature importance	50 features	Conditional inference RF classifier<br><br>50 trees	ACC<br>0.73(95% CI: 0.45, 0.91)

Park et al. 2021 [71]	Glioblastoma	2	N1 = 86<br>N2 = 41	Concurrent chemoradiotherapy/ radiotherapy	Recurrent glioblastoma<br><br>Radiation necrosis	3T MRI<br>T1W1<br>T1C<br>T2WI<br>ADC	N4 bias correction<br>Signal normalization<br>Resamples 1x1x1mm3	Pyradiomics<br>263	F-score<br>Lasso<br>MI	**LASSO features**<br>first-order_kurtosis<br>First-order_mean_absolute_deviation.<br>First-order_range<br>GLCM_inverse_difference_normalized.<br>GLCM_informal_measure_of_correlation1<br>GLCM_informal_measure_of_correlation2<br>GLRLM_run_variance.<br>GLSZM_gray_level non-uniformity<br>GLSZM_low gray level zone emphasis<br>GLSZM_size zone non-uniformity<br>GLSZM_small area high gray level emphasis<br>NTGDM_complexity<br>NTGDM_strength<br>Shape_flatness<br>Shape_major axis length<br>Shape_maximum 2D diameter (slice)<br>Shape_mesh volume<br>Shape_sphericity	KNN<br>SVM<br>AdaBoost	LASSO feature selection + SVM<br>AUC – 0.80 (0.65-0.95)
Li et al. 2023 [72]	High-grade glioma	1	162	Surgery, postoperative adjuvant temozolomide chemoradiation	6th, 9th, 12th, 15th, 18th month progression and recurrence	3T MRI<br><br>T1WI, CET1WI, T2WI, FLAIR	Histogram normalization 0-255<br>Resampling 0.5x 0.5x3mm3	Pyradiomics<br><br>2344 features	Mann-Whitney test<br>LASSO	-	KNN<br>RF<br>SVC, fully connected network FCN	FCN<br>Radiomics Model<br>AUC = 0.77<br><br>Clinical + Radiomics = 0.78
Hettal et al. 2020 [73]	Brain oligometastasis	1	20	SBRT	Radionecrosis vs Progression	1.5 or 3T<br><br>MRI<br><br>CE-T1WI	Discretized no of bins to 8 using BitDepthRescale_Range<br><br>Resampling	IBEX software<br><br>1766 features	Fisher score<br>ReliefF<br>T-score<br>chi-score<br>Wilcoxon<br>Gini index<br>Mutual information maximization	-	Decision tree<br>Bayesian, discriminant analysis, nearest neighbor, neural network, partial least square and principle component regression, random forest, SVM, bagging and boosting	Bagging<br>AUC 0.83(0.65-1)

Table S2: Nasopharyngeal Carcinoma

First Author	Cancer site	No of centers	Sample size	Treatment modality	Outcomes	MRI (Magnetic Field)	Image Preprocessing	Radiomics features extractor software; no of features	Feature selection method	Features used	Predictive Model	Model Evaluation results
Li et al 2022 [74]	Nasopharyngeal Carcinoma	1	156	IMRT NTZ treatment	PFS	3 T MRI<br><br>T1WI, Proton Density, DCE-MR	K^{trans}(efflux rate constant), K_{ep} (reflux rate constant)<br><br>V_e (the extracellular extravascular volume)<br><br>V_p (intravascular plasma volume fraction)<br><br>Maps were extracted from the DCE-MR images.	AK Software<br><br>360 features	Z-score normalization<br><br>PCC<br><br>Univariate Cox regression ($p<0.05$)<br><br>LASSO Cox regression	**K^{trans} features:** Long Run Emphasis_AllDirection_offset1_SD, Long Run Emphasis_angle0_offset1, Angular Second Moment, Low Level Gray Level Emphasis_AllDirection_offset_SD, Low Run Low Gray Level Emphasis_angle0_offset1, High Intensity Small Area Emphasis, Small Area Emphasis<br><br>**V_e features:** Long Run Emphasis_AllDirection_offset1_SD, Long Run Low Grey Level Emphasis_AllDirection_offset1_SD, Small Area Emphasis, Low Intensity Area Emphasis, Uniformity, High Intensity Small Area Emphasis<br><br>**K^{trans} + V_e features:** Ktrans_Long Run Emphasis_AllDirection_offset1_SD, Ktrans_Cluster Prominence_angle90_offset1, Ve_Long Run Emphasis_AllDirection_offset1_SD, Ktrans_Long Run Emphasis_angle0_offset1, Ktrans_Angular Second Moment, Ktrans_Long Run Low Gray Level Emphasis_angle0_offset1, Ve_Long Run Low Level Gray Level Emphasis_AllDirection_offset_SD, Ve_Low Intensity Small Area Emphasis, Ve_Small Area Emphasis, Ve_uniformity, Ve_High Intensity Small Area Emphasis, Ktrans_High Intensity Small Area Emphasis, Ktrans_small Area Emphasis<br><br>**Clinical variables:** Clinical stage, T stage, treatment with nimotuzumab	Radscore and Clinical Variables by Multivariate Cox Regression<br><br>Kaplan-Meier Analysis	**Combined Radscore**<br><br>**K^{trans} + V_e + Clinical** = 0.732 (95% CI 0.599-0.864)<br><br>Nomogram cutoff score = 3.1)<br><br>High-risk (n= 38 24.36%)<br>Low risk(n = 118, 75.64%)
Du et al. 2019 [30]	Nasopharyngeal Carcinoma	2	N1 = 277<br>N2 = 60	IMRT Concurrent or adjuvant	PFS<br><br>3-year disease progression	3T MRI<br><br>CE-T1WI<br>T2WI	Resampled spatially 1x1x4mm^3	Pyradiomics<br><br>525 features	PCC<br>ICC<br>Hierarchical Clustering	**Clinical**: T stage, Overall stage<br>**Radiomic**: shape sphericity, CE-T1W first order mean absolute deviation.<br>CE-T1W wavelet LL GLCM sum entropy.<br>CE-T1 W wavelet LL GLRLM GLNUN	Support vector machine	Radiomic + Clinical AUC = 0.80 (95% CI: 0.73, 0.89)

				chemotherapy								
Zhao et al. 2020 [75]	Locally advanced nasopharyngeal carcinoma	1	123	Induction Chemotherapy (IC)	IC response and survival<br><br>Stable disease<br>Progressive disease<br>Partial response<br>Complete response<br>IC responder (CR/PR)<br>Non-responder SD/PD	MRI<br><br>T1W1, T2WI<br>CE-T1WI	-	MATLAB<br><br>4503 features	Two-sample t-test<br>LASSO	Nineteen features were selected.	SVM with linear kernel	Clinical +Radiomics nomogram<br>C-index = 0.863<br><br>IC responder 3-year PFS = 84.81%

Table S3: Hepatocellular/Liver Cancer

First Author	Cancer site	No of Centers	Sample size	Treatment modality	Outcomes	MRI (Magnetic Field)	Image Preprocessing	Radiomics feature extractor software; no of features	Feature selection method	Features used	Predictive Model	Model Evaluation results
Chen et al. 2023 [31]	Hepatocellular carcinoma	1	144	Trans arterial Chemoembolization TACE	Complete response Partial response Progressive disease Stable disease	MRI<br><br>1.5 T 3.0T<br><br>Axial T2-weighted sequence mDIXON-T1WI	Resampled 1x1x1mm^3 Fixed bin width 25	Pyradiomics<br><br>440 features	ICC Handpicked, mRMR(KNN, SVM)	-	KNN<br>SVM LASSO DNN	DNN<br>AUC = 0.796<br><br>Clinical + DNN<br>ACU = 0.735
Liu et al. 2022 [76]	Hepatocellular carcinoma	2	144<br><br>N1= 94<br>N2=46	Trans arterial Chemoembolization	Tumor response and survival	1.5T MRI<br><br>T1W1, T2WI, DWI, CE-T1WI<br><br>3.0T<br>T1WI, T2WI, CE-T1WI	-	Pyradiomics<br><br>1210 features	mRMR<br>LASSO	T2_original_shape_Maximum2DDiameterRow<br>T2_wavelet.LHL_firstorder_Median<br>T2_wavelet.LLL_firstorder_Kurtosis<br>ap_wavelet.HLL_firstorder_Skewness<br>ap_wavelet.LLH_glrlm_ShortRunEmphasis<br>ap_original_glcm_Imc1<br>pvp_original_shape_Maximum2DDiameterRow<br>pvp_original_glcm_Idn<br>pvp_original_shape_Flatness<br>dp_original_shape_Flatness<br>dp_wavelet.LLH_glszm_LargeAreaHighGrayLevelEmphasis<br>dp_wavelet.HHL_glszm_SmallAreaLowGrayLevelEmphasis<br>T2_wavelet.LHL_firstorder_Median<br>T2_wavelet.HLL_ngtdm_Busyness<br>dp_original_glcm_Imc1	Univariate and Multivariate Logistic regression	Clinical Model AUC 0.609<br><br>Radiomics model AUC 0.754<br><br>Combined AUC 0.781
Bodalal et al. 2023 [77]	Colorectal liver metastasis	1	112	Liver resection	Tumor Hypoxia	3.0 T MRI<br><br>T2WI<br>DWI<br>ADC	-	Pyradiomics<br><br>4032 features	Chi-square<br>Correlation without outcome<br><br>RF<br><br>Linear regression	Features selected by at least four out of seven feature selection techniques.	**DWI b200**<br>Logistic Regression with Decision Tree<br><br>**DWI ADC**<br>Gaussian Naive Bayes with Logistic Regression	**Radiomics signatures**<br><br>DWI b200 AUC = 0.79 (95% CI: 0.61-0.93)<br><br>DWI ADC AUC = 0.72 (95% CI: 0.50-0.90)

									Logistic regression<br><br>Recursive feature selection<br><br>Light gradient boost machine			
Shahveranova et al. 2023 [63]	Colorectal Carcinoma Liver Metastases	1	42	Microwave ablation	Local Tumor Progression	1.5 T<br><br>T2 fat suppresses (phase 2) and early arterial phase T1 weighted fat suppressed (Phase 1)	z-score normalization	Olea Sphere Software<br><br>111 features	Spearman correlation analysis LASSO	**Phase 1**<br>Intercept, shape sphericity, shape elongation, shape flatness, first order energy, first order interquartile range, GLSZM large area low gray level emphasis, GLDM gray level variance<br><br>**Phase 2**<br>Intercept, Shape sphericity, shape elongation, shape flatness, first order energy, first order 10th percentile, first order range, first order skewness, first order kurtosis, GLCM cluster shade, GLCM correlation, GLRLM short run emphasis, GLRLM gray level nonuniformity normalized, GLSZM size zone nonuniformity.<br>GLSZM low gray level zone emphasis, NGTDM coarseness, GLDM gray level variance, GLDM large dependence low-level emphasis	LASSO<br>Logistic Regression	Clinical Model AUC 0.887 (0.807-0.967)<br><br>Clinical +phase1 AUC 0.927(0.860 – 0.993)<br><br>Clinical + Phase 2 0.981 (0.948 – 0.999)

Table S4: Breast Cancer

First Author	Cancer site	No of centers	Sample size	Treatment Modality	Outcomes	MRI (Magnetic Field)	Image Preprocessing	Radiomics features extractor software; no of features	Feature Selection Method	Features used	Predictive Model	Model Evaluation Results
McAnena et al. 2022 [78]	Breast cancer	1	74	Neoadjuvant Chemotherapy	Complete pathological response pCR	MRI 1.5T	FBN 32<br>Resampled $2x2x2\ mm^3$<br>Lagrangian interpolation	LIFEx<br>61 features	LASSO	Discretized kurtosis<br>NGTDM contrast<br>GLSLM short zone grey level emphasis<br>GLZLM zone percentage	SVM	**Radomic model** AUC 0.753<br>**Radiomics + estrogen receptor status** AUC 0.811

Table S5: Other cancer sites

First Author	Cancer Site	No of Centers	Sample Size	Treatment Modality	Outcomes	MRI (Magnetic Field)	Image Preprocessing	Radiomics Features Extractor Software; No of Features	Feature Selection Method	Features Used	Predictive Model	Model Evaluation Results
Wang et al. 2021 [79]	Locally Advanced Rectal Cancer LARC	1	183	Neoadjuvant Chemotherapy	Good/Poor responders PFS	Two MRI<br><br>3.0 T<br><br>T2WI<br>DWI<br>CE-T1WI (1 pre, 3 post)	Resampling 1x1x1mm^3 Gaussian filtering with sigma values 0.5, 1.0, 1.5	3D Slicer<br><br>942 features	mRMR<br>LASSO	T2_original_GLSZM_Large Area Emphasis, T2_log_sigma_0.5mm_3D_GLCM_JointEntropy, CE_T1WI_original_shape_SurfaceArea, CE_T1WI_original_shape_MajprAxisLength, CE_T1WI_log_sigma_1.5mm_3D_GLRLM_LongRunGrayLevelEmphasis, CE_T1WI_log_sigma_1.5mm_3D_GLCM_Autocorrelation, CE_T1WI_log_sigma_1.0mm_3D_GLRLM_ShortRunEmphasis, CE_T1WI_log_sigma_1.0mm_3D_GLDM_Dependence Variance, CE_T1WI_log_sigma_5.mm_3D_GLSZM_GrayLevelNonUniformityNormalized, CE_T1W1_log_sigma_0.5.mm_3D_GLDLM_SmallDependenceEmphasis CE_T1Wl_log_sigma_0.5.mm_3D_GLCM_JointAverage ADC_original_GLRLM_LowGrayLevelRunEmphasis ADC_log_sigma_0.5.mm_3D_glrlm_LongRunLowGrayLevelEmphasis	LR<br>RF<br><br>Nomogram	**LR** Radiomics AUC = 0.842(95%CI: 0.741-0.943)<br><br>**Nomogram** AUC = 0.898 (95%CI: 0.819 - 0.978)
Fang et al. 2020 [80]	Locally Advanced Rectal Cancer LARC	1	120	Concurrent Chemotherapy and radiation therapy CCRT	Treatment response<br><br>**Three Classes** Complete response<br><br>Partial response<br><br>Progressive disease	3.0 T MRI<br><br>Sagittal T2WI, axial T1WI, axial T2-FS, DWI ADC,	Normalized image intensity	In-house MATLAB 2017a code<br><br>1026 features	PCC<br>LASSO	Sagittal_T2_GLRLM_LongRunHighGrayLevelEmphasis, Axial_T1_GLRLM_RunPercentage, Axial_F2-FS_LL_GLRLM_ShortRunLowGrayLevelEmphasis, AxialDWIb0_GLRLM_LongRunHighGrayLevelEmphasis, AxialDWIb800_GLRLM_LongRunEmphasis, AxialDWIb800_ShortRunLowGrayLevelEmphasis, ADC_GLCM_Variance, Sagittal_T1_GLRLM_RunLengthNonuniformity, Axial_T1_HH_GLRLM_RunPercentage, enhancedMRI_SurfaceArea, enhancedMRI_LLH_Skewness, Coronal_T1_GLCM_dissimilarity, enhancedMRI_GLCM_homogeneity	RF<br>LR<br>SVM	RF AUC on test set = 0.798 (95% CI: 0.678-0.917)

Jajodia et al. 2021 [33]	Uterine Cervical Cancer	1	52	chemoradiation	Clinical prognostications<br><br>Recurrence<br><br>Distant metastasis<br><br>lymph node metastasis<br><br>FIGO stage	1.5 T MRI<br><br>DWI	ADC maps calculations<br><br>PreADC (ADC1)<br>PostADC (ADC2)<br>ChangeADC	Pyradiomics<br><br>851 features	PCC	**Radiomics**<br>original_gldm_SmallDependenceLowGrayLevelEmphasis<br>original_glszm_LowGrayLevelZoneEmphasis<br>original_glszm_SmallAreaLowGrayLevelEmphasis<br>wavelet_HHH_glcm_ClusterShade<br>wavelet_LLL_gldm_DependenceEntropy<br>wavelet_LLL_firstorder_Uniformity<br>wavelet_LLL_glrlm_GrayLevelNonUniformityNormalized<br>wavelet_HLL_glszm_GrayLevelVariance<br>wavelet_HLH_gldm_SmallDependenceHighGrayLevelEmphasis<br>wavelet_HLH_firstorder_Median<br>wavelet_HLH_glszm_GrayLevelVariance<br>wavelet_HLH_glszm_SmallAreaHighGrayLevelEmphasis<br>wavelet_HHH_glszm_ZoneEntropy<br>wavelet_HHL_glrlm_RunEntropy<br>wavelet_HHL_glszm_GrayLevelVariance<br>wavelet_HHL_glszm_ZoneEntropy<br>original_firstorder_Energy<br>original_firstorder_TotalEnergy<br>wavelet_LHH_firstorder_TotalEnergy<br>wavelet_HHH_glszm_LargeAreaHighGrayLevelEmphasis<br>wavelet_HHL_firstorder_TotalEnergy<br>wavelet_LLL_firstorder_Energy<br>wavelet_LLL_firstorder_TotalEnergy<br>original_gldm_DependenceEntropy	Monotone Multi-layer perceptron neural network<br><br>KNN	**Recurrence**<br>Radiomics +ADC1<br>AUC = 0.8<br>Kappa value = 0.55<br><br>**Metastasis**<br>Radiomics + ADC1 + ADC2 + ChangeADC<br>AUC = 0.84<br>Kappa = 0.65<br><br>**FIGO Stage**<br>Radiomics + ADC1 + ADC2 + ChangeADC<br>AUC = 0.71<br>Kappa = 0.25<br><br>**Lymph Node**<br>Radiomics + ADC1 + ADC2 + ChangeADC<br>AUC = 0.75<br>Kappa = 0.6
Speckter et al. 2022 [81]	meningiomas	1	93	Gamma knife radiotherapy	Response to radiosurgery	3T MRI<br><br>T1WI<br>CE-T1WI	-	Pyradiomics<br><br>337 features	LASSO	**Radiomics**<br>LoG_sigma_1.0mm_3D_firstorder,InterquartileRange, logarithm_NGTDM_Busyness<br><br>**Non-Radiomic**<br>Karnofsky performance status	Radiomics Score	Combined model AUC = 0.88
Yang et al. 2021 [82]	Vestibular schwannoma	1	336	Gamma Knife Radiosurgery	Tumor Regression (Without Pseudoprogression, With Pseudo regression)<br><br>Tumor Non-Response	1.5 T MRI<br><br>T1WI, T2WI, CE-T1WI	Resampling 0.50 x 0.50 x 3.00 mm^3<br><br>T1WI and T2WI are co-registered on CE-T1WI	MR Radiomics Platform<br><br>576 features	Two sample t-tests with Bonferroni correction<br><br>LASSO	**Tumor regression vs non-response**<br>T2W- Histogram Standard deviation, T2W-LLL-Texture-GLRLM long run emphasis,<br>CE-T1WI_ LLH Texture-LBP-Mean of LBP,<br>CE-T1WI_ HLL-Texture LBP_Uniformity of LBP,<br>CE-T1WI_ LLL Histogram Minimum<br><br>**Tumor regression with and without pseudoprogression**<br><br>CE-T1WI_Tecture_GLRLM Long run low gray level emphasis.	SVM with radial basis function and Bayesian optimization	CASE 1<br>AUC = 0.913<br><br>CASE 2<br>AUC = 0.881

							Z-score normalization			CE-T1WI_Histogram Skewness, T1WI-LLH-Texture-GLCLM Cluster Tendency, T2WI_Texture-GLCLM Cluster tendency, T2WI_HLL Histogram Range		
Siow et al. 2022 [83]	Locally advanced Hypopharyngeal Cancer	1	198	Concurrent Chemoradiotherapy	Overall survival and PFS	3T MRI<br><br>Post-contrast T1	Low-frequency intensity non-uniformity was removed using the N4 bias correction function.<br><br>Normalization of intensity-signal intensity that differs more than 3 std was removed, resampling 1x1x1mm^3.<br><br>FBN 64	Pyradiomics 744 features<br><br>Wavelet filters LLL, LLH,LHL,LHH, HLL, HLH, HHL and HHH	ICC<br><br>PCC<br><br>Univariate Cox analysis<br><br>LASSO Cox regression	**Overall survival**<br>log.sigma.1.5.mm.3D_firstorder_90Percentile<br>log.sigma.1.mm.3D_firstorder_Energy<br>log.sigma.1.mm.3D_firstorder_TotalEnergywavelet-LHL_glszm_SizeZoneNonUniformity<br>**Progression-free survival**<br>log.sigma.1.5.mm.3D_firstorder_90Percentile<br>log.sigma.1.5.mm.3D_glcm_SumEntropy<br>log.sigma.1.mm.3D_firstorder_Energy<br>log.sigma.2.mm.3D_glcm_SumEntropy<br>log.sigma.2.mm.3D_ngtdm_Busyness<br>original_glszm_SmallAreaEmphasis<br>wavelet-LHH_glszm_SizeZoneNonUniformityNormalized<br>wavelet-LHL_glszm_SizeZoneNonUniformity<br>wavelet-LLL_glcm_Imc1<br><br>**Clinical:** T4a stage, T4b stage, N2c stage	Multivariate Cox regression model	**Overall survival**<br><br>Combined radiomic–clinical model iAUC = 0.671(95%CI: 0.637-0.693)<br>**PFS**<br>Combined model iAUC = 0.675 (95% CI: 0.641-0.687)
Liu et al. 2022 [84]	Pediatric medulloblastoma	2	N1 – 226 (113 training, 113 test set)<br><br>N2= 27	Surgical resection	PFS and OS	3T MRI<br><br>T1w and CE-T1w	Normalized between 0-225	In-house program<br><br>1294 features	PCC<br>LASSO Cox regression	CE-T1WI_ori_shape compactness<br>CE-T1WI_ori_shape_max_diameter<br>CE-T1WI_ori_fos_Energy<br>CE-T1WI_ori_glszm_LZSE<br>CE-T1WI_ori_glszm_HGLSE<br>CE-T1WI_Coif3_glszm_SZHGE<br>CE-T1WI_Coif4_fos_Root_mean_square	Radiomics signature<br><br>Multivariate cox regression	Radiomics signature PFS<br>C-Index on external test set = 0.717 (95% CI: 0.657-0.777)<br><br>Radiomic Nomogram +clinicopathological factors C-index on external test set = 0.677(95% CI: 0.638-0.743)
Lin et al. 2023 [34]	Endometrial cancer	4	A = 337<br>BCD = 84<br>Training = 235<br>Validation = 102	-	Recurrence-free survival	1.5T or 3.0T<br><br>CE-TW1<br>T2W	Resampling 1mm3 via the B-spline curve interpolation	PyRadiomics<br><br>1072 features	ANOVA<br>LASSO<br>Wilcoxon rank sum test	**Clinicopathological**<br>Histopathological type, grade, MILVSI, FIGO stage<br><br>**Region IA**<br>original_glcm_Correlation CE -T1W1<br>original_glszm_ZoneEntropy_ CET1W1	XGBoost<br><br>FM - fusion model that combined clinicopathological factors and	FM (IA)<br>AUC =<br>0.85 (0.77–0.93)<br><br>FM (IA with 3 mm PA)<br>AUC=0.83 (0.73–0.92)

	External testing =84	z-score normalization	wavelet-HHH_gldm_DependenceVariance_CET1W1,<br>wavelet_LLL_firstorder_energy CET1W1<br>wavelet-LLH-glszm-largedependence high gray level emphasis CET1W1<br>original shape Least axis length T2W1<br>wavelength LLH_glszm size zone nonuniformity T2W1<br><br>**IA with 3 mm PA**<br>original_shape_Flatness CE-T1WI, original_shape_LeastAxisLength CE-T1WI, original_gldm_LargeDependenceHighGrayLevelEmphasis CE-T1WI, original_glszm_ZoneEntropy CE-T1WI, wavelet-LLH_firstorder_Skewness CE-T1WI, wavelet-LLL_firstorder_Maximum CE-T1WI<br><br>**IA with 5 mm PA**<br>original_shape_LeastAxisLength CE-T1WI, original_glszm_ZoneEntropy CE-T1WI, wavelet-LHL_firstorder_Skewness CE-T1WI, wavelet-HLL_gldm_DependenceEntropy CE-T1WI, wavelet-LLL_firstorder_Energy CE-T1WI, wavelet-LLL_firstorder_Maximum CE-T1WI, wavelet-LLL_firstorder_TotalEnergy CE-T1WI, original_shape_Maximum2DDiameterRow T2WI	radiomics features.<br><br>IA-intratumoral area<br><br>PA, peritumoral area;	FM (IA with 5 mm PA) AUC =0.83 (0.72–0.92)
